# Attitudes toward artificial intelligence and robots in healthcare in the general population: a qualitative study

**DOI:** 10.3389/fdgth.2025.1458685

**Published:** 2025-01-27

**Authors:** Paulina Smoła, Iwona Młoźniak, Monika Wojcieszko, Urszula Zwierczyk, Mateusz Kobryn, Elżbieta Rzepecka, Mariusz Duplaga

**Affiliations:** ^1^ Department of Health Promotion and e-Health, Faculty of Health Sciences, Institute of Public Health, Jagiellonian University Medical College, Krakow, Poland; ^2^ Department of Epidemiology and Population Studies, Faculty of Health Sciences, Institute of Public Health, Jagiellonian University Medical College, Krakow, Poland

**Keywords:** artificial intelligence, robots, health 4.0, in-depth interviews, qualitative content analysis

## Abstract

**Background:**

The growth of the use of artificial intelligence (AI) and robotic solutions in healthcare is accompanied by high expectations for improved efficiency and quality of services. However, the use of such technologies can be a source of anxiety for patients whose expectations and experiences with such technology differ from medical staff's. This study assessed attitudes toward AI and robots in delivering health services and performing various tasks in medicine and related fields in Polish society.

**Methods:**

50 semistructured in-depth interviews were conducted with participants of diversified socio-demographic profiles. The interviewees were initially recruited for the interviews in a convenience sample; then, the process was continued using the snowballing technique. The interviews were transcribed and analyzed using the MAXQDA Analytics Pro 2022 program (release 22.7.0). An interpretative approach to qualitative content analysis was applied to the responses to the research questions.

**Results:**

The analysis of interviews yielded three main themes: positive and negative perceptions of the use of AI and robots in healthcare and ontological concerns about AI, which went beyond objections about the usefulness of the technology. Positive attitudes toward AI and robots were associated with overall higher trust in technology, the need to adequately respond to demographic challenges, and the conviction that AI and robots can lower the workload of medical personnel. Negative attitudes originated from convictions regarding unreliability and the lack of proper technological and political control over AI; an equally important topic was the inability of artificial entities to feel and express emotions. The third theme was that the potential interaction with machines equipped with human-like traits was a source of insecurity.

**Conclusions:**

The study showed that patients' attitudes toward AI and robots in healthcare vary according to their trust in technology, their recognition of urgent problems in healthcare (staff workload, time of diagnosis), and their beliefs regarding the reliability and functioning of new technologies. Emotional concerns about contact with artificial entities looking or performing like humans are also important to respondents' attitudes.

## 1 Introduction

The definition of artificial intelligence formally proposed in the 1980s says that it “refers to the science and engineering of making intelligent machines, through algorithms or a set of rules, which the machine follows to mimic human cognitive functions, such as learning and problem solving” (1). The transformation of healthcare resulting from the introduction of artificial intelligence (AI) is associated with expectations for the improvement of personalized care, the optimization of medication dosages, the enhancement of population health management, the implementation of clinical guidelines, the support of patient education, and providing assistance with virtual health assistants (2). Many authors believe that the use of AI in healthcare may ameliorate supply-and-demand challenges that are present in many healthcare systems, manifesting in workforce shortages and inequities in access to care (3).

It seems that health professionals present overall positive attitudes toward the use of AI in healthcare; however, their opinions depend on the field of medicine in which they are involved (4). Health professionals perceive AI more as a challenge and a new opportunity to provide care, so they expect more education, training, and clear legal guidance on the responsibilities of using AI (4).

Differences in the attitudes of healthcare professionals toward AI depend on the field of medicine. For example, mental health practitioners are rather skeptical about the possibility of substituting a psychotherapist with AI to improve the results of therapy (5). The review by Bitkina et al., who analyzed studies published from 2011 to 2021, showed that the main areas of research on AI applications were oncology (55%), pulmonology (19%), cardiovascular medicine (9%), and orthopedics (8%) (6).

Society's and patient's views on the use of AI in healthcare systems play a crucial role in its widespread adoption (2). The research assessing the general public's attitudes toward AI has yielded mixed results. People's opinions largely depend on the extent to which AI is integrated into specific healthcare roles, such as whether AI is intended to replace human healthcare professionals or merely assist them. Nevertheless, both patients and physicians generally perceive the AI-physician relationship as a type of synergy (7).

The area of AI application also matters (8). A survey performed in the USA before the COVID-19 pandemic revealed that more than 55% of respondents believed that AI would make healthcare better or somewhat better (9). The opposite opinion was expressed only by about 6%, and 19% were undecided (9). The respondents' attitudes differed depending on the type of clinical application. For example, the use of AI for reading chest radiographs was accepted to a decidedly greater degree than AI for making cancer diagnoses. According to the report published by the Pew Research Center in 2023, fewer than 40% of Americans would feel comfortable if their healthcare provider relied on AI for their medical care and believed it would lead to improved patient outcomes (10).

Sociodemographic characteristics have a significant impact on attitudes towards the use of AI in healthcare. A study involving patients and their companions at a tertiary hospital in Germany revealed that around 54% of participants held a positive or very positive view of AI in healthcare, while only 5% had negative opinions (11). Acceptance levels were notably lower among older patients, women, individuals with lower educational attainment, and those with limited technical proficiency.

Society exhibits varied opinions on the use of AI, marked by a degree of uncertainty. Individuals and patients recognize the potential advantages, yet they also express concerns about possible risks and ethical implications.

A qualitative study conducted by Čartolovni et al. revealed that patients anticipate AI could shorten waiting times and lessen administrative tasks in healthcare (7). When discussing the advantages of AI in skin cancer screening, patients highlighted the potential for faster diagnostics and improved access to healthcare (12). A systematic review focusing on multi-stakeholder preferences regarding AI implementation in healthcare found that patients and the general public mainly expect benefits such as enhanced test accuracy, a reduction in medical errors, decreased workloads for healthcare professionals, lower healthcare costs, better access to care, and shorter wait and travel times (4). In turn, health professionals foresee increased efficiency resulting in reduced clinical and non-clinical workloads, time savings, improved workflow efficiency, enhanced medical capabilities through fewer errors, heightened quality of clinical skills, and better risk detection. Some studies also pointed to advancements in decision-making and recommendation systems (4).

Tran et al. observed that respondents who held negative views towards the integration of AI and wearable devices in healthcare emphasized several concerns, including the insufficient replacement of human intelligence, the risk of hacking, and the potential misuse of private patient data (13). Notably, the segment of staunch opponents to these technologies was relatively small, accounting for only 3%, in contrast to the 20% of respondents who believed that the benefits significantly outweigh the associated risks.

The willingness of older adults to share personal health information when engaging with AI-enabled caregiver robots was found to be influenced by trust, privacy concerns, and levels of social isolation (14). Furthermore, mixed sentiments were evident among members of the general public regarding the necessity of consent for the utilization of personal health data in AI research (15). It appears that such decisions are contingent upon the manner in which the data is utilized and the entities responsible for its use. A systematic review conducted by Vo et al. reinforced that while the general public and patients are amenable to sharing anonymized data for AI development, they harbor significant reservations about sharing data with insurance and technology firms (4).

Ethical considerations frequently emerge in conversations surrounding the integration of AI and similar technologies within the healthcare sector. Ploug et al. conducted an analysis of public perceptions regarding AI-assisted decision-making in healthcare (16). Participants in this study articulated that they anticipated physicians would retain ultimate responsibility for treatment decisions. Beyond this expectation, they underscored the importance of both the explainability of decisions made by AI and the necessity for prior testing to identify potential discrimination within the system. Insights gleaned from focus groups with patients revealed various concerns related to the safety of AI, the potential erosion of patient autonomy, biases in data sources, and data security issues (17).

The topic of explainability continues to spark extensive debate in the context of AI application in healthcare. Amann et al. stress the imperative for a multidisciplinary approach that, from the patient's viewpoint, examines the interaction between human practitioners and medical AI (18). Additionally, participants in other studies have voiced apprehensions regarding the potential loss of decision-making authority if AI is employed in patient-centered care (19).

It seems that the attitudes of the general audience are influenced by their familiarity with AI technologies, as well as their medical histories. A qualitative study conducted by Pelly et al. indicated that prior negative encounters with AI were a significant source of concern among patients with a history of myocardial infarction (20). Conversely, patients who had experienced diagnostic errors previously expressed a greater appreciation for the advantages of AI-based symptom checkers (21). Additionally, individuals with a personal history of melanoma demonstrated stronger support for the integration of AI in medicine compared to those with negative histories (22).

Cultural context plays a significant role in how AI is perceived within the healthcare sector. Patients frequently express the view that AI lacks the ability to offer emotional support or empathy concerning their condition (7, 19). Additionally, another study revealed that patients considered human interactions to be more critical to their care than AI interventions (23). Consequently, patients demonstrate a greater willingness to accept the implementation of AI for health-related tasks that do not involve the physician-patient relationship, such as scheduling appointments or follow-up communications (19).

The perception of robots in healthcare and related areas, e.g., social robots, is closely associated with attitudes toward AI use (4, 11). Advanced robotic solutions used in healthcare usually rely on some type of AI to provide support to the users. A close relationship between robotics and AI in healthcare can be observed in surgery, rehabilitation, management, and support tasks (24). Also, telemedicine-based systems enabling medical triage, diagnostics, surgical and non-surgical treatment, and specialty care benefit from their combined application (24). The use of AI and robots brings us to the actual realization of the “4P Medicine” model (25), postulating that a new degree of quality in healthcare will be substantiated by predictive, personalized, preventive, and participatory attributes.

The perspectives of Polish society regarding the use of AI in healthcare have not been extensively examined. Our research represents the initial phase of a larger effort to evaluate citizens’ views on the implementation of innovative technologies in health service delivery. Prior to formulating a strategy for the quantitative aspect of the research project, a series of interviews was conducted to inform the design of survey studies. The main aim of this study was a qualitative analysis of the attitudes of representatives of Polish society toward the use of AI and robots in healthcare and related areas, e.g., elderly care. We have conducted fifty in-depth interviews asking the participants about various aspects of the use of information systems in healthcare, including a set of questions about the perception of AI and robotic systems. The interviewees' responses to the latter set of questions were analyzed and presented in this paper.

## 2 Material and methods

The interviews were part of a mixed-method research project that analyzed the acceptance and use of e-health and health 4.0 technologies in Polish society. The qualitative part of the project aimed to understand various motives related to using e-health, including attitudes toward AI and robotic solutions in healthcare. The study was conducted in line with the Consolidation Criteria for Reporting Qualitative Research (COREQ, see Supplementary Table S1) (26). The research material consisted of 50 semistructured qualitative interviews. The interview guide comprised open-ended questions that explored various topics. These included the interviewees' overall attitudes towards new technologies and their experiences with technology-related anxiety, their use of the Internet to access health information, and their understanding of telemedicine and e-health. Additionally, the guide examined the utilization of e-health applications, perceptions of the barriers and facilitators affecting the use of e-health services, and personal experiences and opinions regarding remote consultations with physicians. Questions about the application of AI and robots in different healthcare and social care contexts were integral to the interview guide. This paper presents an analysis of the interviews in part related to the use of AI and robots in these areas. The interview guide is provided in Supplementary Table S2. The interviews were conducted between October 2023 and January 2024 in participants’ homes or via the Microsoft Teams application. After each interview, summary notes were made. The research team consisted of a sociologist, a nurse, a public health specialist, a nutritionist, and a research assistant with a background in archival sciences. All members had experience in conducting qualitative interviews.

The research obtained approval from the Jagiellonian University Bioethical Committee (Decision No 1072.6120.296.2022, issued on January 18, 2023). Research participants were informed about the study's aims and had to sign an informed consent form before the interview began. All interviews were recorded, transcribed, and analyzed with MAXQDA Analytics Pro 2024 software. The transcripts of the interviews were not returned to the interviewees. The interviewees were not invited to familiarize themselves with an analysis either; however, they were asked to provide their e-mail addresses so they could be sent the article presenting the findings based on the interviews.

To gain a sufficiently deep insight into the research material, basic assumptions of qualitative content analysis (QCA) were deployed (27). QCA is a method of analyzing qualitative data concentrated on building analytical categories based on careful reading and successively going to higher levels of abstraction, from initial familiarization with the material to derivation of latent content from it (28). IM and PS performed the analysis in the following stages:

1. Familiarizing themselves with the data - reading the interviews to know all of the material; at this stage, researchers noted first insights on important topics that came up, which were checked in the next phases.
2. Dividing up the text into meaning units and condensing them - as the study was based on interviews, some of the meaning units were just answers to the questions. In other cases, when the answers were more developed and complex, they were meaningful fragments within those answers.
3. Formulating codes to label the meaningful units - condensing the text into meaningful units requires an analytical description; codes sum up the meaning of the given unit and provide analytical insight. The codes were derived inductively. Their number changed during the research process. The coding tree is available in Supplementary Table S3.
4. Developing categories – codes were grouped according to their meaning, meaning that categories consist of codes that refer to the same topic (like Fear of robots/AI in medicine or Attitude toward robot-assistant of the elderly). Categories and building codes were the basis for analytical interpretation.

**Figure 1:**
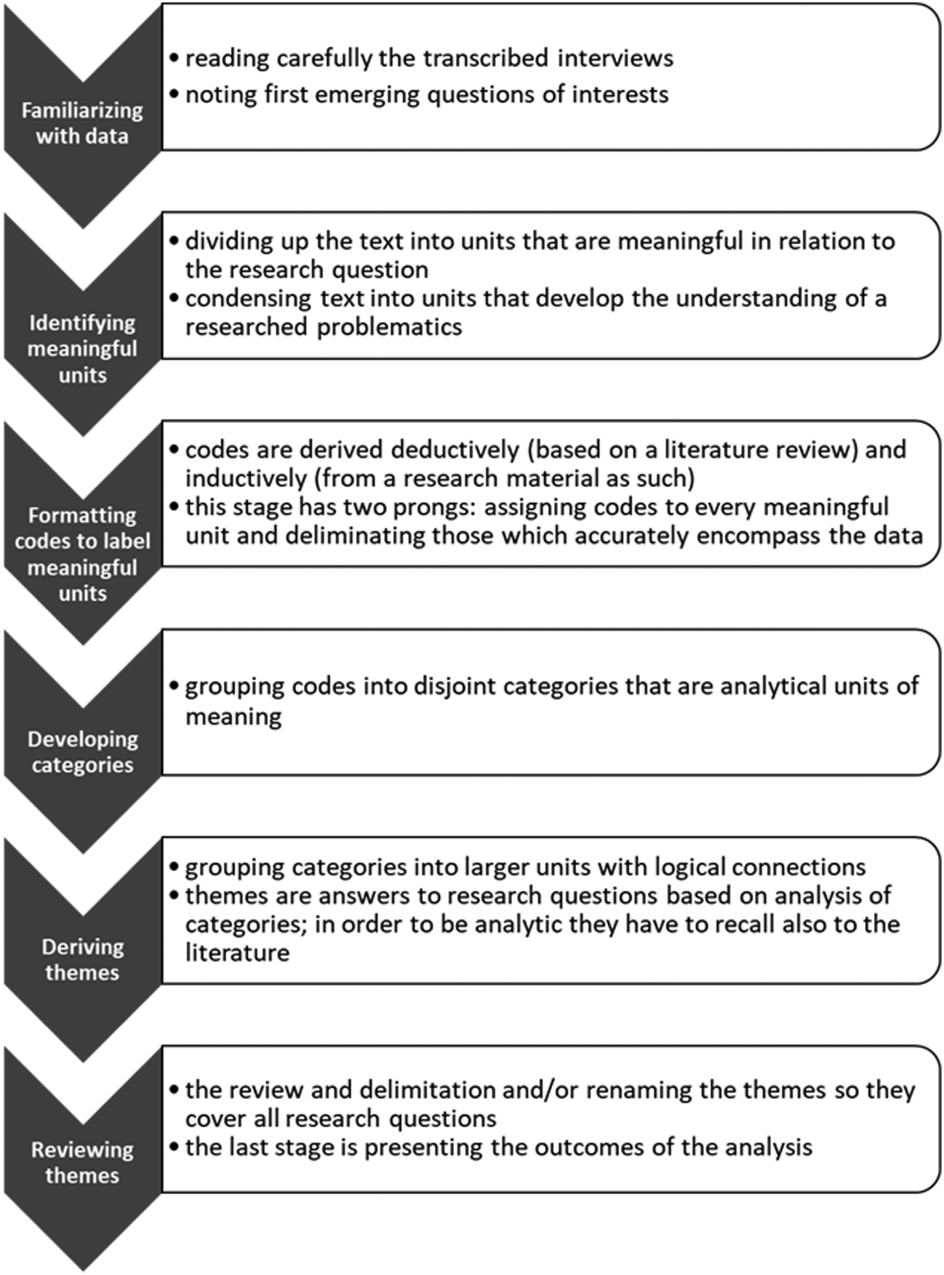
The stages of the analytical process in thematic analysis.

An interpretive approach was used to assess the answer to the research question. Interpretive description in health sciences is used in studies that are focused on understanding patients' experiences or complex views (29, 30). In this research, we used it to better understand interviewees’ attitudes toward robots and AI in healthcare and social care. The interpretive approach allowed us to concentrate on how the respondents themselves understand the issue and what is important for them to accept those solutions. The emphasis in the analysis was not on individual attitudes (which would have to be measured somehow) but on the elements of attitudes in the statements. This allowed us to interpret the phenomenon that some of the interviewees had generally negative attitudes to the use of AI or robots but simultaneously believed that there were areas where they would significantly improve the effectiveness of treatments. The stages of the analytical process in thematic analysis are presented in Figure 1.

## 3 Results

### 3.1 Participants' characteristics

Interviewees with diverse socio-demographic characteristics (age, gender, place of residence, education, type of job) and time spent on online activities and use of new technologies were recruited for the study. Each participant was interviewed only once. Initially, interviewees were recruited for convenience, and then a snowball technique was used. After the first interviews, any problems arising during the initial phase of the study were reviewed, and the usefulness of the interview guide was discussed. Some interviewees required clarifications on key terminology related to the use of information technologies in healthcare. It was also suggested that examples of potential applications, such as AI, should be included to enhance understanding of the topics discussed. However, the changes to the interview's structure were not significant. Fifty persons (26 women and 24 men) participated in the qualitative study. The mean age of the participants (standard deviation, SD) was 43.8 (14.2), with a range of 18–76 years. Detailed characteristics of the group are shown in Table 1.

**Table 1:** Characteristics of the study group.

Variable	Categories of variable	%	*n*
Sex	Female	48.0	26
	Male	42.0	24
Place of residence	Rural	48.0	24
	Urban <100,000 population	12.0	6
	Urban of 100.000–500,000 population	8.0	4
	Urban >500,000 population	32.0	16
Education	No education	2.0	1
	Lower than secondary	26.0	13
	Secondary or post-secondary	48.0	24
	University degree	24.0	12
Vocational status	Public sector employee	76.0	38
	Retired	8.0	4
	University or college student	4.0	2
	Unemployed/inactive	12.0	6
Marital status	Single	34.0	17
	Married	56.0	28
	Widowed/divorced/separated	10.0	5
Chronic disease	No	88.0	44
	Yes	12.0	6
State of health	Excellent	14.0	7
	Very good	34.0	17
	Good/satisfying	48.0	24
	Unsatisfying	4.0	2

### 3.2 Themes

As the analysis was conducted according to qualitative content analysis guidelines, three main themes, covering the main dimensions of interviewees' attitudes towards artificial intelligence (AI) and robots in healthcare and related areas, emerged as a result. The themes answer the research question by highlighting the dimensions of AI/robots in healthcare that seemed the most important for interviewees. Two of them are the positive and negative aspects of using AI and robots in medicine. The third one focuses on ontological concerns about AI that exceed the fears and objections regarding the usefulness of the technology. The ontological concerns revealed that human-robot interactions were, in some cases, hard to accept because they undermined common sense assumptions of what defines human beings.

#### 3.2.1 Theme 1. Positive attitudes towards AI and robots in health care

##### 3.2.1.1 Subtheme A. Greater accuracy during surgery and in diagnostics

It appears interviewees are positive about using robots in surgery, as they believe that robots can perform complex operations that humans cannot due to physical limitations.

###### 3.2.1.1.1 Precision during surgical operations

According to the interviewees, using robots during surgery could enable less invasive and/or more complex procedures.

Researcher:

Do you think, for example, that a robot could replace a surgeon during surgery?

Interviewee:

But it's already happening, I think. I think it's already happening. There are certainly big specialized devices of some kind that also make it possible to carry out operations in such a bloodless way, especially when something happens in the sinus area. I think they're also just doing things like that on the heart, they're putting some of these things in, watching on monitors. (PSm_10_2.01.2024, Item 705-708)

Other responses to the same question as above:

This is, in my opinion, the future, and robots will be more in industry and in medicine; there will be more of them, and there will be complex procedures that the human hand will not do. (PSm_6_28.12.2023, Item 387)

Not all things will be done as precisely by a human as by a robot, and that should be rather, so science should move in that direction. (MWoj_1_ 21.10.2023, Item 436)

Although I think there are areas, for example, of very precise things like eye surgery or things like that, where probably a robot would ideally be more accurate and more precise than a human. (MK_3_12.10.23, Item 181)

###### 3.2.1.1.2 More accurate diagnosis of medical images

The development of AI means that it can be used to interpret the result of imaging techniques, such as magnetic resonance imaging (MRI) and computer tomography (CT) scans, with greater accuracy than specialists can.

I think that with such a kind of intelligence, instead of a human being to describe it, if you upload such AI a lot of it, it would sweep there. So, it would be more effective than a doctor. I think yes, it would be more effective for describing different images of injuries or different diseases like tomography, and there are these images, tumors, not so, because a human being cannot always see a millimeter there or there, and AI would. Well, I think it would make a revolution… (EB_4_06.12.2023, Item 462)

###### 3.2.1.1.3 Faster diagnosis of difficult-to-treat or diagnose diseases

According to the interviewees, using AI could speed up the diagnostic process of certain diseases. Some interviewees claimed that sometimes patients receive an accurate diagnosis only after a long journey between specialists in different fields, or the time needed to reach a final diagnosis is extremely long, and using AI could significantly shorten it. They expect that using AI could save time and enable a more accurate diagnosis, which, in effect, could result in prolonging life or effectively curing a disease.

I have heard opinions on this that it would certainly shorten the period of diagnosis because it would be able to analyze the data faster than a human being and spit out, in inverted commas, this diagnosis. (MWoj_3_29.12.2023, Item 260)

###### 3.2.1.1.4 Holistic diagnosis and fewer mistakes

According to the interviewees, AI-based diagnostic systems will be able to diagnose diseases faster and solve more complex medical problems outside the capacity of health professionals. Moreover, AI or robots will not make mistakes, as they are not limited by the senses and can process more information at once than the human brain can handle.

Because a human cannot do everything, and here, well, maybe this AI will help in a much bigger way, faster, maybe easier, maybe more accurately, so it seems to me at least. (MWoj_2_29.12.2023, Item 273)

##### 3.2.1.2 Subtheme B. Relieving the burden on medical staff and medical registrars

Excessive responsibilities and emotional strain can translate into inefficient work among medical staff. From the interviewees' point of view, humanoid robots in the roles of assistants or staff could be an apt solution to improve the functioning of healthcare.

###### 3.2.1.2.1 Robot in the role of a physician assistant

Such a role for a robot received a positive response among the interviewees. In their opinion, doctors already have assistants, so it would be possible for a robot to be such an assistant, and it would not make them uncomfortable to communicate with the robot instead of a human being during an appointment. Moreover, robots could guide doctors in diagnosis and treatment and take care of patients' medical records. This would allow the doctor to perform their duties more efficiently and reduce fatigue. However, the interviewees emphasized that they only accepted the robot as a physician's assistant, not a doctor.

Researcher:

And do you think a robot in the role of a doctor's assistant would work?

Interviewees:

Well, doctors have their own assistants anyway; it doesn't matter if it is a robot. (PSm_6_28.12.2023, Item 372-376)

In the role of a doctor's assistant, it's quicker because, after all, it's an assistant, and the doctor has sort of control over it; there are things like, I don't know, hard-to-reach places where, well, somewhere a robot can actually be cleverer. I don't know and get into places where it's difficult to access, in that respect, yes. (EB_5_8.12.2023, Item 490)

###### 3.2.1.2.2 Robot in the role of a nursing assistant

Interviewees also expressed positive attitudes towards a robot as a nursing assistant. The work of nurses, especially in a hospital ward, requires precise organization of duties, taking into account emergencies. Hence, the robot could facilitate and relieve the nursing staff of routine tasks such as temperature checks and blood pressure measurements. In addition, as the robots do not get tired, they can care for patients more effectively, for example, by delivering medication.

Researcher:

Do you think, for example, such a robot in the role of, say, a nurse, or could it work? (MWoj_3_29.12.2023, Item 270-271)

Interviewee:

That's what I was thinking, I don't know; such a driving robot could just drive up with a tray, and the patient would take these medicines. So that would be some kind of relief from that nurse's workload. (MWoj_3_29.12.2023, Item 272)

###### 3.2.1.2.3 Robots in the role of medical registrar

Interviewees agreed on the implementation of this humanoid robot role. According to the interviewees, the formalities accompanying admissions to hospitals or other medical facilities are cumbersome. The registration staff is often overwhelmed by their workload. Hence, implementing a robot as a medical registrar would make the registration process more effective. Interviewees list such arguments as “robots will not start unnecessary conversations,” “they will be less susceptible to patients’ emotions,” and “they will be more efficient (concise and quick).” Some interviewees also claimed that they believe that with robots, the registration process will be fairer (everyone will have an equal chance to register). And robots will ensure equal courtesy toward patients.

Researcher:

Do you think, for example, that such a robot in the role of admissions staff could work?

Interviewees:

Such a registration robot has a constant view of all the bases and can look at all the appointments at any time and accommodate many people at once, so this would speed up the work. (MK_5_19.11.23, Item 372)

I don't talk to the robot about it, it just registers, so I think it's fine. I have no problem saying my name and surname details, it would certainly be nicer than what some of the ladies in the clinics do, it wouldn't have to, and it wouldn't give its opinion. (EB_3_4.12.2023, Item 463)

It could be because the registrations would be fair because sometimes they sign up by acquaintance, it would just be by order. (PSm_7_28.12.2023, Item 393)

Interviewees often agreed that medical staff are overburdened with excessive tasks that AI or robots could perform. This would minimize the risk of mistakes due to staff fatigue and increase the efficient use of their working time.

Researcher

What are the main advantages, in your opinion, of using robots in medicine and healthcare?

Interviewees:

And fatigue as well, for example, well, if you actually work for a few hours a day, you can actually be tired as a human being, and a robot like that, well, it doesn't feel fatigued, it doesn't feel any feelings, so it would just be able to actually work all the time so you know some kind of a link that drives it would be replaced all the time, that’s fine. (EB_10_19.12.2023, Item 407)

Purely theoretically, as a robot, it shouldn't make mistakes, and it doesn't feel stressed, so it should do it accurately, but in practice, I'm not quite sure about that. (MK_5_19.11.23, Item 384)

The doctor may be tired, but the robot may not. The doctor might make a mistake not because he got the wrong data but because he didn't hear something. Or his brain didn't notice something. A robot shouldn't be like that. And so on. You could go on like that for a long time from here. (MK_8_06.12.23, Item 165)

###### 3.2.1.2.4 Care for the elderly (with disabilities)

Caring for the chronically ill elderly requires much effort and even sacrifice on the side of formal or informal (most often, family) caregivers. It can also be uncomfortable for an older person due to signs of dependency or the inability to carry out daily activities. Some interviewees liked the idea of implementing a robot caregiver for an older person, seeing it as a way to help minimize the burden on caregivers and also as a chance to provide more effective care services.

Interviewees who were asked about opinions regarding robot assistants for older people who live alone showed mixed attitudes. As positive aspects of such a solution, some pointed out that contact with robots can be less stressful in intimate activities, like washing or changing diapers. Robots will also not lose their temper and will always be as patient and polite as needed. Nevertheless, for some interviewees, a robotic assistant is acceptable only in situations when an elderly patient is unconscious or deprived of any social and family relations.

For people who are alone, this would be a good solution, in the case of unconsciousness, this would be good. For elderly, solitary people it is as much as possible such a thing. (PSm_2_15.10.2023., Item 296)

It is worth mentioning that interviewees also stressed that the decision to use robots should be made independently by the elderly patients.

#### 3.2.2 Theme 2. Negative attitudes towards AI and robots in healthcare

##### 3.2.2.1 Lack of human qualities affects the tasks performed

According to respondents, a lack of human qualities such as emotions and empathy can lead to ineffective work due to the inability to understand patients' needs or reactions to some medical procedures.

On the other hand, I do not know whether such a robot would know how to take responsibility for this person, for his emotions. (PSm_10_2.01.2024, Item 722)

It seems to me that the doctor is not going to be replaced because here, too, we need some element of empathy, well, just human contact. Some people just need to feel sorry for themselves. (MWoj_3_29.12.2023, Item 268)

For some interviewees, the ability to feel emotions and empathize with others is an essential part of the treatment process.

So, however, these emotions are human warmth, which is always this advantage over such a robot, if only. (UZ_10_31.12.2023, Item 405)

##### 3.2.2.2 The unreliability of technology

As AI is based on technology and information systems, according to the interviewees, this can result in technical problems during surgery or diagnosis. Here are arguments and examples given by the interviewees, mirroring those from the previous point.

###### 3.2.2.2.1 During surgical procedures

The unreliability of AI during surgical procedures and emergencies, e.g., sudden cardiac arrest, was one of the most common concerns.

Researcher:

And do you think robots could replace a doctor in the operating room, for example? (MWoj_3_29.12.2023, Item 278)

Interviewee:

Here, too, to some extent, well, because here I'm not sure if, at least as I say, that's how technology has developed so far or if every robot would have as smooth a movement as a human hand as a human wrist. They always seem to me to be sort of like that, more mechanical and jerkier with those movements. (MWoj_3_29.12.2023, Item 280)

Interviewee:

I'm going to be operated on by a robot, well this doctor who is able to react suddenly, differently let's say that this robot is programmed.. (MK_6_19.11.23, Item 231)

###### 3.2.2.2.2 In diagnosing patients

Interviewees showed mixed attitudes towards AI diagnosing patients. On the one hand, it could be a time-saver. Still, on the other hand, AI-based diagnostic systems, according to the interviewees, are not yet fully prepared to make diagnoses autonomously.

And what do you think of the use of AI, for example, to make diagnoses? Diagnoses of, for example, a given disease or the determination of treatment methods? (PSm_10_2.01.2024, Item 650)

Diagnostics, I don't know. You know what, really, how many of these images would have to be there, or how many of these cases would have to be described for the computer to be able to cope with diagnosing difficult diseases? (PSm_10_2.01.2024, Item 652)

###### 3.2.2.2.3 Lack of public or political control over the algorithm

Interviewees also feared mistakes in the algorithm on which an AI-based information system is built. This could lead to abuse (deliberate actions of the developers) or lead to unreliability due to errors or insufficient skills on the part of the programmers.

Researcher:

And do you think that AI can be trusted? (MK_7_19.11.23, Item 322)

Interviewee:

That they will help, well as you know it's a machine, there could be a mistake. Wrong setting. It's known that it can harm, but a human being, also, if they're mentally tired, they can also harm a sick human being. There are no perfect things or perfect robots or perfect people. The end. (UZ_1_15.10.2023, Item 389)

Interviewee:

Due to the fact that it is, however, AI, it is just some algorithm, a program, something like that created, admittedly by a human, but it is just a machine, a thing, a certain algorithm, which can also make a mistake, break down or mislead. (MK_7_19.11.23, Item 328)

##### 3.2.2.3 Laziness/reducing mental effort

The facilities offered by AI are making it more and more commonplace, even at the doctor's office. As a result of the interviewees' experience, it appears that doctors are also using Internet browsers, etc., during their visit, which is perceived negatively by the patients. Some research participants interpret using devices to check something during their visit as a sign of insufficient medical knowledge.

Researcher:

What other disadvantages do you see to the use of AI?

Interviewee:

I think that's what it is. And I've sort of encountered more than once that a doctor has looked something up on the internet during a consultation with me. (MK_1_8.10.23, Item 434)

Moreover, according to the interviewees, this is developing on such a large scale that people may stop thinking and making decisions independently in the future. Some claim that using AI-based apps or GPS maps makes people lazy and dependent on technology.

No. These new technologies fool people rather than help people. You can see it in children who can't live without a phone in their hand from an early age. Such children are not creative. It blocks some thinking. (UZ_5_29.12.2023, Item 34)

AI can help, but at the same time, it can make people stop thinking a bit on their own. (UZ_6_29.12.2023, Item 189)

##### 3.2.2.4 Anxiety about high economic costs

There were also concerns about the financial costs of producing or using robots in medical settings.

Researcher:

And do you see, do you have any other concerns just related to the use of robots and AI in medicine and healthcare? Any more concerns?

Interviewee:

At this stage of simply developing this AI, this technology, we are not yet able to introduce these solutions on such a large scale. Well, these are also terribly expensive solutions. So, I don't know if there will be enough funding. (MWoj_3_29.12.2023, Item 288)

#### 3.2.3 Theme 3. Ontological concerns

The third theme concerns substituting human qualities and artificial intrusion into relationships.

##### 3.2.3.1 Fear of displacement of human work

Among the concerns raised by interviewees was the fear of replacing human beings (doctors in particular) with AI by broadening the scope of tasks done by AI or robots to the point of the total expulsion of people from their work. First, interviewees' apprehension is connected with taking jobs - something in which they have specialized and which should ensure income for them. Another was more ontological and connected with an aversion to contact with machines instead of humans.

Researcher

And are we doomed to replace doctors with AI systems?

Interviewees:

I hope not. And I think there needs to be more of a focus on young doctors being able to learn, to develop so that they can actually treat patients reliably. Of course, with the use of modern technologies, but I think they cannot replace the doctor and this direct contact with the patient. (EB_6_12.12.2023, Item 506-509)

I think no, I wouldn't want a robot. I would like a human being. (PSm_4_27.12.2023, Item 373)

##### 3.2.3.2 Anxiety about the non-human intelligence

Interviewees spoke about the features that define a human being vs. a machine – feeling emotions, empathy, independent thinking, and the ability to take responsibility for their actions – and had negative attitudes towards possible human-robot interactions. Their anxiety results from a fear of “confusion” between human and artificial entities, which can endanger social relationships.

According to some interviewees, a robot should never have a human appearance or voice.

Some of the interviewees claimed to highly value interpersonal relationships and a sense of empathy from the other person. They argued that such relationships could never be achieved with non-human artificial entities.

According to another group of interviewees, robots should look different than people so as not to “pretend” (and thus replace) them.

Researcher:

And would you feel comfortable talking to such a robot? (MWoj_1_ 21.10.2023, Item 405-406)

Interviewee:

His movements didn't suit me, somewhere I would be disturbed by his movements, I would be disturbed by his voice because he certainly wouldn't have a voice like a human because I, unless I just have a different idea of a robot that .. moves like a robot, not like a human. (MWoj_1_ 21.10.2023, Item 412)

Some interviewees stressed the importance of the human capacity to feel and show empathy.

It's just an advantage that some people - like this interpersonal context - at work they talk to each other, and when you come to work and you just see a soulless machine flying around and saving people just like you do, but also actually, on the one hand, it's good that this kind of work for example in a hospital affects people a lot. If they see, for example, someone dying, like someone is terribly ill, even if it's not family, and the robot does what it can and flies on. The anesthesia - such a robot. (EB_9_19.12.2023, Item 519)

Well, I am rather against the humanization of machines, devices. Let a robot be a robot. A human being, let’s be a human being, and this boundary I think, should not be somehow lost. (MWoj_7_06.01.2023, Item 233)

##### 3.2.3.3 Fear of being dominated or assaulted by machines

It appears that interviewees' fear of AI or robots is related to the belief that AI-based systems can get out of control and – in a way, purposely– attack humans. They spoke about the threat of assault on individual persons, for example, during an operation or a kind of machine rebellion, when robots or AI will take control over all cyber systems.

I read something somewhere once. So, at the moment, .., out of sci-fi films, or it's like someone has watched different films, a lot of, for example, directors are already introducing such motifs that, for example, the robots are out of control. (EB_10_19.12.2023, Item 371)

We'll get to some absurdities, like these science fiction films, some Elysium we'll create, I don't know, where computers will be everything, they'll decide everything, even who is to survive. (PSm_10_2.01.2024, Item 678)

Because of sci-fi movies I have a fear of AI, maybe this. (MK_5_19.11.23, Item 352-353)

## 4 Discussion

Interviewees view the potential for more precise surgeries and expedited diagnostics as the primary driver of the acceptance of AI and robotics in healthcare. Previous research has underscored the significant promise of AI in enhancing the quality of healthcare services and accelerating diagnostic processes (31, 32) The ability of AI to perform intricate surgeries, such as sinus and cardiovascular procedures, marks a notable advancement in the medical field, according to the interviewees. They believe that AI will enable surgeries that surpass human capabilities.

While the interviewees acknowledged the increasing prevalence of AI-assisted surgeries globally, their insights are largely derived from media and online sources rather than personal experiences. They posit that the swift diagnostic capabilities of AI could lead to more effective treatments for challenging illnesses like cancer. Notably, research institutions are already developing algorithms for cancer diagnosis (33, 34). Imaging tests present another area where AI-based diagnostics excel. Interviewees expressed confidence in the AI system's capacity to analyze data and deliver diagnoses faster than their human counterparts, which they found its crucial merit, as they perceived diagnosis duration to be essential for successful treatment.

The efficiency and accuracy of AI-driven diagnostics have been validated in prior studies (35). So, the interviewees' opinions reflect the outcomes of ongoing studies. It also seems that they expect an improvement in medical procedures. These sentiments are particularly pronounced among individuals grappling with chronic illnesses (31). In their quest for improved healthcare quality, interviewees affected by illnesses place their faith in the potential of AI technology. Their expectations are further incited by the reports on the successful integration of AI in medical diagnostics of various conditions, including skin cancer and neurological disorders (36).

It appears that the interviewees showed positive attitudes towards the use of AI and robots as physician assistants. However, they value contact with the doctor and underlined a greater sense of security. Hence, this acceptance only leans towards the use of robots as physician assistants. Interviewees said that doctors have – and need – assistants, so they would not mind if they were robots. Furthermore, they believed a robot could provide treatment guidance and minimize the risk of erroneous treatment choices. It seems that broader audiences are aware of the risks, confirmed by research, pointing to the unfavorable effects of an unreliable memory and/or incomplete knowledge of a given field of medical practice (37, 38).

The nursing staff also attracted much attention from the interviewees. The interviewees often referred to situations in which the nursing staff is overloaded due to excessive duties, and using robots as assistants could reduce their workload and make it more efficient. It appears that general opinions are in line with the results of earlier studies in this context (39, 40). It should also be noted that the interviewees' experience using medical services also led to a desire to replace admissions staff with robots. The belief that with the implementation of an admissions robot, check-in procedures would be faster, without unnecessary delays, was present in the responses of the majority of interviewees. In the context of robots' advantages, the risk of stress and fatigue among medical staff, which affects their performance at work, was also highlighted (we discuss it more broadly in the next point).

Due to the lack of emotions, AI-based robots can become more emotionally effective than humans. In the eyes of the interviewees, the use of robots to care for elderly and solitary people is associated with benefits not only directly for these people but also for their caregivers. Research on this kind of care for older people has been ongoing for years, and it has been shown to improve the well-being of older people, but also to have benefits for their caregivers, helping them better manage their time and help with physically difficult and dirty tasks (41–43). In our study, the majority of interviewees were within the age group of potential caregivers. Previous research has indicated that elderly individuals who interact with humanoid robots generally exhibit more favorable attitudes toward these robots compared to caregivers (32). This may explain why the interviewees emphasized the potential use of robots primarily in situations where elderly individuals are living alone or are unresponsive.

Notably, the respondents highlighted that artificial (non-human) caregivers should be considered a last resort, particularly when there are no family members or others available to assist with daily caregiving tasks. This perspective appears to be influenced by cultural norms that shape attitudes toward different caregiving approaches, as much as beliefs or knowledge do (44). n Poland, the duty of caring for older individuals is primarily viewed as a family responsibility, regarded as a moral obligation and a aspect of intergenerational solidarity. However, this caregiving obligation is often seen as a burden that can adversely impact various aspects of a caregiver's life (45).

According to the researchers, despite the benefits, such a solution may be a challenge for the elderly due to a lack of adequate competence in using electronic devices (46). Hence, it is necessary to educate the elderly in the use of technology. Notably, the interviewees emphasized that artificial (non-human) caregivers should be a last resort, specifically when no relatives or other people can be engaged in daily caring duties. Attention was also given to the mental health of doctors and nurses. Although interviewees did not want doctors and nurses to be replaced by robots, they believed that through the use of AI, the staff would be less exposed to stress and fatigue, which would minimize the risk of medical mistakes and improve their work comfort.

The fear of a lack of empathy from AI and robots was common among interviewees' concerns. It appears that despite the strong interest in AI and the desire to develop it in healthcare, many concerns still need to be addressed. Showing emotion seems to be an important element that medical staff should display. Patients are not only looking for competent professionals but also for those with developed interpersonal skills (47). Previous studies have shown that patient trust and empathy from medical staff increase patient satisfaction and predict more effective adherence to treatment (47, 48).

Interviewees were also concerned about the unreliability of the technology. Despite expressing approval for robots to perform complex surgeries, the interviewees had concerns about inadequacies in algorithms or adverse events, such as the unreliability of AI in the operating theatre. Criticism about AI is sometimes based on its perception as a “black box”; this may lead to the public's losing trust in AI-based systems (49). The interviewees' worries are mainly related to concerns about developing appropriate algorithms. It is worth noting that there were two opposite views on this problem. One posits that it is such a complicated technology that even programmers do not actually know what they are working with (the “black box”). The other states that AI is just a tool but so complicated that only programmers know how to manage it, which makes it even more vulnerable to human mistakes or bad will.

Some interviewees stated that using new technologies reduces mental effort in everyday situations, which they judged negatively. Concerning contact with doctors, it gave them the impression that medical doctors do not have sufficient knowledge. Those interviewees did not seem to view AI as having any useful functionalities but rather perceived it as a kind of toy or the equivalent of a cheat sheet. In that sense, they interpreted using AI tools as revealing a lack of skills and a moral attitude that should not have a place among representatives of a high social trust profession. This belief can be connected to their limited knowledge of and experience with AI (50, 51).

The last subtheme is connected to the perception of the economic costs of introducing AI and robots. The interviewees pointed out two dimensions of such expenses. One is the cost of production, which they perceived as an obstacle to its wider implementation. Another issue is operating costs, which seemed to the interviewees to be too high for public healthcare settings. It is worth noting that here, the main obstacle is not the emotional attitude toward new technologies but beliefs about the condition of public finances.

At least some study participants perceived robots and AI as entities with a hard-to-define ontological status. Classical (traditional) machines, which most users are used to, have the status of objects, which means they are things controlled by human beings and – in common sense – do not have any shared features with living beings. AI and medical robots seemed to cross the border between objects and living beings in the interviewees' eyes. Some were perceived this way thanks to their appearance (e.g., assistive robots for the elderly), and some were perceived this way due to how they performed (AI). Previous research has shown that human appearance can positively influence trust in robots (43, 52); however, this study revealed opposing attitudes. This could be caused by the fact that the interviewees spoke only about their views and imagination, though they had no real experience with robots or AI (53).

Another issue is the rather vague knowledge of how AI works (54). Furthermore, media and movies present it as a kind of black box that even programmers do not understand fully, which can lead to it getting out of human control. The concerns about human-AI interactions range from worries about the future of workplaces to fear that dark imaginings from sci-fi movies could come true. However, interpreting this as resulting only from a lack of knowledge or experience with AI or robots would be a simplification. This is where explanations drawn from psychological discoveries can provide an otherwise lacking dimension for interpretation. It appears that among interviewees, attitudes toward robots and AI are similar to those toward other human individuals or groups. Such a phenomenon has already been seen in other studies (55, 56).

The negative attitudes especially seem to have the traits of how one perceives a hostile group, which can cause a real threat to the individual. Researchers call these realistic threats and identity threats (55). The first type encompasses fears with a material basis, like threats to physical or material well-being. The second type refers to the sense of one's identity and distinctiveness from others. It is worth noting that these two kinds of threats were originally studied in intergroup relations (57) but have found application in the research on human attitudes toward robots, as people tend to anthropomorphize them (58).

Subthemes A and C, which are distinguished in Theme 3 as “the fear of displacement of human work” and “being dominated or assaulted by machines,” are related to a potential threat to one's safety. Although the concerns discussed above in Theme 2 seem similar (the unreliability of technology), the apparent causes for these concerns differ. Theme 2 is associated with interviewees' fears resulting from the perceived risk of possible human mistakes or violations and a lack of control over them. Theme 3 reflects interviewees' fears arising from the silent assumption that machines either can act intentionally to assault humans or can perform tasks better than them. In some sense, such feelings lead to doubts about the subordinated position of a machine in human-machine interactions and the position of a person as the one who commands other entities (55).

Subtheme B (Anxiety about non-human intelligence) reflects identity threats caused by imagined human-robot or human-AI interaction during which the given technology can act independently or look and behave like a person. The two main reasons for anticipating uncanny and unnerving feelings can be distinguished (59). First, there is a belief in robot/AI agency. It appears that for some interviewees, the feature that distinguishes humans from other animals and entities is the ability to act independently, i.e., decision-making (following one's free will). Some of them also give AI or robots negative characteristics. Future research is needed to examine the connection between those two beliefs. Some insights on the issue indicate that individuals often attribute agency based on minimal cues, such as a partial body shape or movements that can be perceived as intentional (60). As a result, they assign agency and human-like characteristics to objects that resemble humans. The second cause of negative attitudes concerns robots that are visually identical to human beings. The interviewees’ conviction that robots should not look like humans can be interpreted as an ontological fear of being deceived.

It must be noted that some of the interviewees also perceive the lack of human characteristics (i.e., intentionality and agency, which are essential for responsibility) as a possible threat.

The reluctance and negative attitudes towards robots carry an ethical dimension that can influence their acceptance in healthcare settings. Previous studies indicate that attitudes toward technology are linked to its perceived usefulness and the moral attributes attributed to it (61). The attribution of moral qualities and moral judgment is associated with a robot's appearance (human-like robots) and the complexity of tasks they undertake, which may involve some degree of responsibility for their actions. When viewed as moral agents, robots can potentially violate certain moral principles, providing insight into the ambivalence expressed in several interviews. In the context of technology acceptance research, morality can be framed through Jonathan Haidt's Moral Foundation Theory (MFT) (62).

Ho et al. applied Haidt's moral foundations—harm/care, fairness, loyalty, authority, and purity—to create a model for understanding human-machine relationships (63). Their analysis of factors affecting perceptions of emotional AI technologies included constructs derived from MFT (61). In our study, the interviewees' concerns and negative attitudes toward AI and robots reflect these moral dimensions. For instance, the lack of certain human capabilities, like accountability for actions and their outcomes, may be perceived as a breach of the harm/care foundation of MFT. Furthermore, the resistance to robots serving as doctors might be viewed as a challenge to social hierarchy, especially in Poland, where the medical profession is regarded as prestigious and trustworthy. Nonetheless, additional research examining the impact of gender, cultural differences, and real-world interactions with robots and AI in healthcare is warranted.

### 4.1 Limitations of the study

Qualitative studies do not allow for drawing conclusions about population trends; therefore, the results apply only to the research participants - nevertheless, purposeful sampling (as the research team strived to find participants with various socio-demographic characteristics) was used to reflect the diversity of the population and the varied views and attitudes regarding the issues of the study to acquire the most differentiated participants and the broadest scope of views possible. Although the results are in accordance with the literature, there is a possibility that some attitudes prevalent in Polish society are not represented in the study due to the sampling limitations. There is the risk of erroneously interpreting the results, which is why the coder consultations follow Braun and Clarke's rules (64). The decisions on coding, derivation of themes, and interpretation were discussed and included in the study only when both coders agreed on their meaning.

Another possible limitation of the study is that the interviewees, despite answering the questions extensively, have never had any contact with AI or robots. This means that one cannot predict how they would perceive actual interaction. Moreover, it is possible that at least some of them could change their attitude after such an experience, but it would be rather difficult to predict the kind and degree of such a change. Further qualitative and quantitative studies are needed to address the issues mentioned above.

## 5 Conclusions

The study aimed to gain insight into the possible causes of attitudes toward using AI and robots in medical settings. The deployment of QCA and the interpretive approach allowed us to distinguish three main themes encompassing various dimensions of the interviewees’ attitudes. Firstly, positive attitudes were connected with greater trust in technology, recognition of demographic changes (a growing need for care of older people), the conviction that AI and robots can lighten the workload of medical staff, and the belief that technology can perform better in highly stressful or exactingly precise tasks. Secondly, the same characteristics of robots and AI that were seen as advantages were perceived as the opposite by some other interviewees. The lack of emotions or their artificial structures were the main reasons for negative attitudes, like fear or distrust. It appeared that insecurity caused by potential interaction with machines equipped with human-like traits or appearance (Theme 3) is an equally important issue.

Our research examined the intricate attitudes regarding the integration of artificial intelligence (AI) and robots within medical settings. This study provides timely insights into the various factors shaping these attitudes, particularly considering the rising prevalence of AI and robotics in healthcare. The primary themes identified underscore the complex nature of public perception. Positive attitudes are associated with trust in technology and an awareness of demographic shifts that highlight the necessity for elder care. A considerable number of participants acknowledged the potential of AI and robots to address contemporary healthcare challenges, particularly in alleviating the burden faced by medical personnel.

Furthermore, our study affirmed the duality of perceptions surrounding AI and robots in the healthcare sector. While some participants perceive these technologies as beneficial, apprehensions stemming from fear and distrust also surfaced.

An important source of negative attitudes and ethical concerns appears to stem from the perceived deficiency of emotional capabilities in machines. Our findings resonate with the prevailing opinion that interactions between humans and machines frequently lack empathy and emotional intelligence. Many participants emphasized that these qualities are crucial for satisfactory exchanges in healthcare contexts.

Additionally, anxiety related to the discomfort of engaging with humanoid machines emerged as a significant insight in our interviews. This indicates that the design of AI and robots should carefully consider patient expectations. Factors such as enhancing the transparency of AI processes and integrating emotional elements through deliberate design or ensuring human oversight may effectively mitigate these concerns.

We contend that the results of our study yield important insights into the factors influencing attitudes toward AI and robots in healthcare within Polish society. On one hand, individuals acknowledge the potential advantages of technological innovations in reshaping healthcare; on the other hand, numerous apprehensions must be addressed to foster positive interactions and garner support from both patients and the general public when encountering novel solutions.

## Data Availability

The datasets presented in this study can be found in online repositories. The names of the repository/repositories and accession number(s) can be found below: Zenodo https://doi.org/10.5281/zenodo.12627811.
