# Supplementary material for: Attitudes toward artificial intelligence and robots in healthcare in the general population: a qualitative study
Source: Front Digit Health. 2025 Jan 27;7:1458685. doi: 10.3389/fdgth.2025.1458685 (PMC11808042; doi:10.3389/fdgth.2025.1458685)
Supplement: Supplementary file 1 [file Datasheet1.pdf]

Supplementary Material

Table 1. COREQ Checklist

Topic	Item no	Guide Questions/Description	Section of the paper
Domain 1: Research team and reflexivity	1 Interviewer/facilitator	Which author/s conducted the interview or focus group?	2
	2 Credentials	What were the researcher's credentials?	2
	3 Occupation	What was their occupation at the time of the study?	2
	4 Gender	Was the researcher male or female?	
	5 Experience and training	What experience or training did the researcher have?	2
	6 Relationship established	Was a relationship established prior to study commencement?	2
	7 Participant knowledge of the interviewer	What did the participants know about the researcher? e.g. personal goals, reasons for doing the research	2
	8 Interviewer characteristics	What characteristics were reported about the inter viewer/facilitator? e.g. Bias, assumptions, reasons and interests in the research topic	2
Domain 2: Study design	9 Methodological orientation and Theory	What methodological orientation was stated to underpin the study? e.g. grounded theory, discourse analysis, ethnography, phenomenology, content analysis	2
	10 Sampling	How were participants selected? e.g. purposive, convenience, consecutive, snowball	2
	11 Method of approach	How were participants approached? e.g. face-to-face, telephone, mail, email	2
	12 Sample size	How many participants were in the study?	2
	13 Non-participation	How many people refused to participate or dropped out? Reasons?	2
	14 Setting of data collection	Where was the data collected? e.g. home, clinic, workplace	2
	15 Presence of non-participants	Was anyone else present besides the participants and researchers?	2

	16 Description of sample	What are the important characteristics of the sample? e.g. demographic data, date	3.1
	17 Interview guide	Were questions, prompts, guides provided by the authors? Was it pilot tested?	2, Suppl. material
	18 Repeat interviews	Were repeat inter views carried out? If yes, how many?	No
	19 Audio/visual recording	Did the research use audio or visual recording to collect the data?	2
	20 Field notes	Were field notes made during and/or after the interview or focus group?	2
	21 Duration	What was the duration of the inter views or focus group?	2
	22 Data saturation	Was data saturation discussed?	2
	23 Transcripts returned	Were transcripts returned to participants for comment and/or correction	2
Domain 3: analysis and findings	24 Number of coders	How many data coders coded the data?	2
	25 Description of the coding tree	Did authors provide a description of the coding tree?	Suppl. material
	26 Derivation of themes	Were themes identified in advance or derived from the data?	2
	27 Software	Derivation of themes	2
	28 Participant checking	Did participants provide feedback on the findings?	2
	29 Quotations presented	Were participant quotations presented to illustrate the themes/findings?	Yes
	30 Data and findings consistent	Was there consistency between the data presented and the findings?	2
	31 Clarity of major themes	Were major themes clearly presented in the findings?	3
	32 Clarity of minor themes	Is there a description of diverse cases or discussion of minor themes?	4

Table 2. Structured interview guide

1) Health needs and the utilization of healthcare j
2) The attitudes to new technologies and technology anxiety
3) The use of the Internet to access health-related information
4) Contacts with other persons/patients with similar medical conditions via the Internet and social media
5) The knowledge and understanding of telemedicine and e-health
6) The utilization of e-health applications
7) The use of health apps on mobile phone. The need for the use of health monitoring applications
8) The use of e-health applications available for citizens in Poland, e.g., Internet Patient Account
9) Experience with remote physician visits.
10) Barriers to the use of e-health applications. Main sources of anxiety related to e-health
11) Readiness to use remote care in the future
12) The use of other digital services apart from e-health
13) Self-assessed e-health literacy
14) The perception of digital skills of the health professionals
15) Access to electronic medical record
16) The perception of sharing own medical data for research
17) The attitudes to the use of AI in healthcare
18) The attitude to the use of robots in healthcare

Table 3. Code tree

Code tree	Notes	Frequency
Code tree		295
humanoid robot	Does the interviewee think that humanoid robots could check oneself, e.g. as registrars?	0
relief of the nurse	Interviewee claim the robot could relieve the burden on nursing staff.	1
lack of human voice	Interviewee would be disturbed by the fact that the robot does not have a human voice	1
fear of emergencies	Interviewee claim, that a robot wouldn't be able to cope with emergencies	1
does not feel tired	Interviewee claim that the robot would work more efficiently because it does not feel stressed	2
more efficient working	Interviewee claim, that a robot in the role of registrar would serve patients more efficiently	2
aversion to robotic surgery	Interviewee doesn't want a robot to operate on him	1
Not flexible	Interviewee think that the robot, unlike a human being, is not flexible and would not be able to answer non-standard questions	1
Should not look like a human being	Interviewee think, that robots should not look like humans, in order to leave a clear distinction between the human and the artificial	1
No feelings		5
Need to get used to	Interviewee think, that the first innteractions could be strange or unladylike, but he thinks he would get used to them	1
Lack of fear	Interviewee doesn't mention any concerns about i interactions with robots	3
Too expensive	Interviewee afraid that AI/robot solutions may not be implemented in Poland because they are too expensive	1
No empathy	Interviewee think that the downside of robots in registration is that they lack the empathy, the flexibility that humans have (e.g. a human is able to bend the rules a bit to help another, while a robot is not)	3

As a tool	Interviewee think, that a robot could be useful in tasks that are difficult for a human to perform, only being under the complete control of a physician	2
Only simple tasks	Interviewee think, that the robot would be suitable, for example, as a recorder, to perform simple tasks	4
No emotions	Interviewee think, that the robot has the advantage of being emotionless, so that it cannot be led astray by the behaviour of patients	3
complicated operations	Interviewee think, that, thanks to artificial intelligence, operations will be performed that the human hand will not do	21
No mistakes	Interviewee think, that a robot in the role of recorder would not make the mistakes that happen to humans	1
Laugh	Interaction with the robot would make the respondent laugh (funny/stressful)	1
Polite	Interviewee think, that the robot-registrar would always be uniformly polite in behaviour and dress, unlike humans.	4
human organism	Interviewee think, that he would rather talk to a human than a robot	13
fair access	Interviewee think, that a humanoid robot in the role of medical registrar would be fair to patients	1
wrong algorithm	Interviewee think, that AI is based on an algorithm that can be flawed and carry many mistakes	4
the same attitude to each	Interviewee think, that a conversation with a robot in the role of recorder would be concise and quick	2
aversion to the robot	Interviewee would not want a humanoid robot to accompany him when he is lonely	9
physician assistant	badany uważa, że robot mógłby się sprawdzic jako asystent lekarza	20
no remarks	Interviewee believes he would have no problem talking to a robot about his symptoms	4
willingness to talk	Interviewee would like to talk to a humanoid robot	4
fear of AI	Interviewee does not want AI to develop	2

long waiting time		Interviewee think that robot would not work well as a registrar because it would take a long time to register people	1
carer for the elderly			4
	a lot of time spent	Interviewee think, that the robot would have a lot of time for the elderly	1
	positive aspect	Interviewee think, that this is positive and good for carers of eldry people	3
	possibility of interaction	The elderly person would have the opportunity to establish a relationship with the robot	3
	as entertainm ent	Interviewee think, that a robot in the role of caring for an elderly person could work as an "entertainment element"	2
	lack of empathy	Robot, does not show emotion and would not be affectionate towards an eldry person	4
	Prefer time alone	Interviewee think, that himself/herself does not want to have a robot as a companion, because wants peace and quiet and not additional interaction	1
	Hard to imagine	Interviewee can't imagine what it would be like for a robot to help the elderly	1
	Replacem ent of the family	Interviewee recognises the risks of buying a robot instead of family care	1
	For living alone seniors	Interviewee think, that the robot can be a good solution for seniors who do not have relatives or the family cannot take care of them	3
	Making care easier	Interviewee think, that AI and robots are/would be a big help in caring for the elderly	2
	Interaction like with an animal	Interviewee compares interactions with a robot as a companion to interactions with pets	2
	Need to have some emotions	Interviewee think, that a robot should be able to show appropriate emotions, empathy	1

	If looking like a human		1
	Simple tasks	Interviewee think, that a robot can assist an eldry person in situations/tasks that do not require responsibility	3
	Never replace human being		11
	Approval of a senior	Interviewee think, that the consent of the person to be handled by the robot is required	6
	Comfort	Interviewee think, the robot will provide more comfort and less embarrassment to the eldry person.	1
	Patient	Interviewee think, that the robot is better suited to care when the elderly person's patience is required	1
Diagnosis		Interviewee think, that artificial intelligence can diagnose patients and make diagnoses	0
	shorter diagnosis period	Interviewee think, that AI would shorten the period of diagnosis	1
	autonomy of diagnostic systems	Interviewee think, that AI-based diagnostic systems can be fully autonomous	1
	Diseases are to complex	Interviewee think, that the course/symptoms of diseases are too complex to be diagnosed by AI	2
	Believe in	Interviewee would accept the robot/AI diagnosis, but may still have a negative attitude towards technology in medicine	1
	full diagnosis	More precise diagnosis	2
	an error may occur	Diagnostic systems based on artificial intelligence can give erroneous diagnoses	8
	physician supervision	Diagnostic systems based on artificial intelligence should be supervised by a physician	19
	prefers a physician	Interviewee prefer for a physician to give him advice	8
	complete diagnosis	Interviewee think, that a robot could also diagnose and treat a patient	1

Artificial intelligence in the physician's office	What role does the respondent think the physician will have in the event of wider development of artificial intelligence?	0
is for consultation	Interviewee think, that AI could only be used for consultation	1
physician seeks information from AI	Physician during the visit was looking for information about treatment in AI	1
Depends on construction	Interviewee think, that what and if good use of AI in medicine depends on the algorithms or the way it is implemented	3
As learning help for MD	Interviewee think, that AI can only be an aid to further education or a small support for physicians	1
Not enough developed yet		7
It's MD obligation to know how to heal	Interviewee think, that it is a physician's duty to be able to diagnose/know how to treat a patient, as this follows from the very definition of being a physician	2
reluctance to be treated by AI	Interviewee does not want artificial intelligence to treat people	2
guidance on treatment	Interviewee think, that AI could provide guidance in the treatment of diseases	8
fewer mistakes	Artificial intelligence makes fewer mistakes than a physician	9
Acceptance of artificial intelligence in health care	What Interviewees think about the use of artificial intelligence in health care?	0
spirit of the time	Interviewee think, that everything is moving with the times, so he is in favour of	1
Does not get tired	Interviewee think, that the advantage of machines is that they do not tire, unlike humans/fatigue does not affect their work	2
Cheaper	Interviewee think, that robots can in the future be cheaper than employing the right number of people	1

	Does not have an opinion	Interviewee no opinion on AI in medicine, has no knowledge or is not interested in the subject at all	4
	Does not make mistakes like humans		3
	improving health care	Interviewee think, that artificial intelligence will make the health care system work more efficiently	5
	incurable diseases	artificial intelligence will treat untreatable diseases including cancer	3
	facilitation for phycians	Interviewee think, that artificial intelligence makes physicians' work much easier	5
	major advances in medicine		7
	the health care revolution		2
knowledge of the term ‘artificial intelligence’		Can the interviewee explain what he understands by artificial intelligence?	0
	associate with diagnostic systems	Interviewee associates artificial intelligence with diagnostic systems	1
	associates with locators	Interviewee think, that artificial intelligence is mainly "subcutaneous chips" that locate the location of the	1
	phone/smartphone	Interviewee think, that artificial intelligence there is a phone and smartphone	1
	associates with GPT chat	Interviewee associates artificial intelligence mainly with AI-based chat	4
	does not know	Interviewee does not know what is artificial intelligence	2
	robots	Interviewee associates artificial intelligence mainly with robots	3
	knows the term	Interviewee does not know what is artificial intelligence	3
			79
Barriers		What fears the interviewee has about artificial intelligence?	0

Lack of responsibility	Interviewee think, robots would not be responsible	1
Work	Interviewee think for the negative aspect the risk of job losses	1
Out of control	Interviewee is afraid, that AI can get out of control	6
Robots will replace humans	Interviewee is afraid, that robots will take jobs away from humans or even replace humans.	2
May make people lazy	Interviewee think, that AI can make people lazy, e.g. they will stop thinking for themself	2
Unthinkable	For interviewees are inconceivable, e.g. he does not believe in such progress or availability of technological solutions	1
Can not think abstractly	Interviewee think, that a lack of abstraction thinking makes AI unsuitable for specific tasks	1
Unknown rules of functioning and learning	Interviewee think, that we ourselves do not yet know on what principle AI learns or works, and therefore consider its use as risky	3
Input-output	Interviewee think, that the risk is that the AI robot/algorithm only contains what has been written there by humans, which could lead to abuse or poor functioning, or to it not being able to deal with emergencies or unexpected situations	6
Do not think independently	Interviewee think, that the lack of opportunity for si to think and/or gain experience will lead to wrong diagnoses	2
Lack of empathy	Interviewee think, that robots do not have human feelings, empathy and therefore can sometimes harm instead of help	5
Communicatio n problems	Interviewee think, that would have difficulty communicating with the robot/robots insufficiently understand commands.	2
Malfunction	For interviewees the disadvantage of robots is the risk of failure	7
lack of readienss	Interviewee think that society is not ready for the development of artificial intelligence	3

	fear of substitution of physicians	Interviewee is afraid that robots will replace phycians	6
	lack of comfort	Interviewee would feel uncomfortable talking to a robot	7
	lack of emotions	The robot does not show emotions, therefore the interviewee would feel uncomfortable	1
	reluctance to consult	Interviewee would not be convinced by a medical consultations with a robot	5
	lack of trust		18
